# The Spine-Tinker

**Published:** 1829-01-01

**Authors:** 


### LXIII.

### THE SPINE-TINKER—PRESCRIPTION FOR DEVIL'S PUNCH.

"To hear an open slander is a curse.

"But not to find an answer is a worse."—*Dryden.*

The above personage, who, like a true Lincolnshire frog, dived into his native mud to elude the penalties of the law, has lately ventured to approach the surface of certain troubled waters, and issue forth some dolorous croakings from his "*slimy bag.*"* Having himself raked up the story about the exhibition of his portrait in Somerset House, perched between a crooked spine and a big book of forthcoming quackery, he is terribly annoyed by the least allusion to that picture. With his usual *shuffling and tergiversation,* he now endeavours to throw the blame of *bad taste* on his painter—a proceeding in perfect keeping with the spine-tinker's general tactics. We shall therefore drop that subject, and take the liberty of placing in *our* exhibition, for the edification of the profession and the gratification of the said tinker, a self-drawn portrait, which he cannot disown. When in the mood, we can say some severe things ; but no language, of which we are masters, could embody the cutting and the damning satire conveyed in the following autograph :—

℞, 1. *Ammon. Acetat.*

2. Acidi citrici, āā ʒij.

3. Liq. ant. tart. min. xl.

4. *Liq. ammon. acet.*

5. Mucilag. acaciæ, āā ℥j.

6. Tinct. hyosciam. ʒj.

7. Sp. æther. nitrici.

8. Sulph. sodæ, āā ℥ss.

9 Mist. amygdal, ℥iijss.

10. Mist. camphorat. ℥ij.

M. fiat mistura, cujus capiat coch. ij. mag. statim. et quart. repet. horis.

Die August 23, 1825. E. H.†

---

* The designation which the *spine-tinker,* in his "*bolder flight to the general reader,*" gives to the synovial capsules ! !

† Published in the Medical Gazette.

Let the professional reader contemplate the foregoing prescription, (of devil's punch,) produced in a public court of justice, in the year 1828, and now carefully preserved as a specimen of the "march of intellect," in some quarters—let him ask himself and his neighbours where the *acetate of ammonia* is to be found, (for the *fourth* item in the prescription proves that the *liq. ammon. acetat.* is not meant,)—let him imagine (God forbid he should feel) the tormina of the young lady's bowels when she swallowed a mixture containing two drachms of *unneutralized* citric acid!—Let him mark all these things, and admire the *modesty* of the spine-tinker, when he talks of "*his* academical education—an advantage which Dr. Johnson never enjoyed"—a modesty only equalled by the spine-tinker's *mendacity* in accusing Dr. Johnson of *surreptitious* titles. Let him *recollect*, that the spine-tinker, whose auto-pictorial likeness we have hung up above, is the self-appointed defender of the Edinburgh graduate against the monopoly of the London College, and who, on the day of trial, doffed his diploma, disguised him as a bone-setter, and concealed himself behind the crooked spine of a hapless damsel, whose dorsal region he had been knuckling and rubbing with bears' grease for three whole years!

---

and now in the possession of the College of Physicians.—*Ed.*

